# SARS-CoV-2 Susceptibility and *ACE2* Gene Variations Within Diverse Ethnic Backgrounds

**DOI:** 10.3389/fgene.2022.888025

**Published:** 2022-04-27

**Authors:** Nirmal Vadgama, Alexander Kreymerman, Jackie Campbell, Olga Shamardina, Christiane Brugger, Genomics England Research Consortium, Alexandra M. Deaconescu, Richard T. Lee, Christopher J. Penkett, Casey A. Gifford, Mark Mercola, Jamal Nasir, Ioannis Karakikes

**Affiliations:** ^1^ Department of Cardiothoracic Surgery and Cardiovascular Institute, Stanford University, Palo Alto, CA, United States; ^2^ Department of Pediatrics, Division of Cardiology, Stanford School of Medicine, Department of Genetics, Stanford School of Medicine, BASE Initiative, Betty Irene Moore Children’s Heart Center, Lucile Packard Children’s Hospital, Palo Alto, CA, United States; ^3^ Cardiovascular Institute and Department of Medicine, Stanford University, Palo Alto, CA, United States; ^4^ Department of Stem Cell and Regenerative Biology, Harvard University, Cambridge, MA, United States; ^5^ Division of Life Sciences, Waterside Campus, University Drive, University of Northampton, Northampton, United Kingdom; ^6^ NIHR BioResource, Cambridge Biomedical Campus, University of Cambridge, Cambridge, United Kingdom; ^7^ Department of Molecular Biology, Cell Biology and Biochemistry, Brown University, Providence, RI, United States; ^8^ Genomics England, London, United Kingdom

**Keywords:** SARS-CoV-2, COVID-19, ACE2, variants, eQTLs, genetics

## Abstract

There is considerable variability in the susceptibility and progression for COVID-19 and it appears to be strongly correlated with age, gender, ethnicity and pre-existing health conditions. However, to our knowledge, cohort studies of COVID-19 in clinically vulnerable groups are lacking. Host genetics has also emerged as a major risk factor for COVID-19, and variation in the ACE2 receptor, which facilitates entry of the SARS-CoV-2 virus into the cell, has become a major focus of attention. Thus, we interrogated an ethnically diverse cohort of National Health Service (NHS) patients in the United Kingdom (United Kingdom) to assess the association between variants in the *ACE2* locus and COVID-19 risk. We analysed whole-genome sequencing (WGS) data of 1,837 cases who were tested positive for SARS-CoV-2, and 37,207 controls who were not tested, from the UK’s 100,000 Genomes Project (100KGP) for the presence of *ACE2* coding variants and extract expression quantitative trait loci (eQTLs). We identified a splice site variant (rs2285666) associated with increased ACE2 expression with an overrepresentation in SARS-CoV-2 positive patients relative to 100KGP controls (*p* = 0.015), and in hospitalised European patients relative to outpatients in intra-ethnic comparisons (*p* = 0.029). We also compared the prevalence of 288 eQTLs, of which 23 were enriched in SARS-CoV-2 positive patients. The eQTL rs12006793 had the largest effect size (d = 0.91), which decreases ACE2 expression and is more prevalent in controls, thus potentially reducing the risk of COVID-19. We identified three novel nonsynonymous variants predicted to alter ACE2 function, and showed that three variants (p.K26R, p. H378R, p. Y515N) alter receptor affinity for the viral Spike (S) protein. Variant p. N720D, more prevalent in the European population (*p* < 0.001), potentially increases viral entry by affecting the ACE2-TMPRSS2 complex. The spectrum of genetic variants in *ACE2* may inform risk stratification of COVID-19 patients and could partially explain the differences in disease susceptibility and severity among different ethnic groups.

## Introduction

Severe acute respiratory syndrome-coronavirus 2 (SARS-CoV-2) is a novel positive-strand RNA virus identified as the cause of the coronavirus disease 2019 (COVID-19). It is responsible for a pandemic that has cost the lives of over 5.96 M people since the first documented case in Wŭhàn, China, in December 2019 (Zhang and Holmes, 2020).

Disproportionate hospitalisations, deaths, and complications from SARS-CoV-2 in some minority groups suggest there might be a potential biological underpinning driving risk disparities in COVID-19 patients. Although many health ministries have identified age and chronic conditions, such as obesity, diabetes, cancer, and immunodeficiency, as major drivers of risk, genetic markers of susceptibility have not yet been used as a metric for risk (Booth et al., 2021).

The most well-known and evaluated mechanism of SARS-CoV-2 infection is the binding and uptake of viral particle through the human angiotensin-converting enzyme 2 (ACE2) receptor, a type 1 integral membrane glycoprotein (Tipnis et al., 2000). This process is initiated by the trimeric transmembrane spike (S) glycoprotein protruding from the viral surface. During infection, the S protein is cleaved into subunits, S1 and S2. S1 contains the receptor-binding domain (RBD), which allows the virus to directly bind to the peptidase domain of ACE2, while S2 is responsible for membrane fusion upon viral infection (Lan et al., 2020; Zhang and Holmes, 2020). Thus, coding variants within *ACE2* could potentially alter SARS-CoV-2 binding and possible infection responses in different individuals based on host genetics. Host genetics are important in susceptibility to other viral infectious diseases, including SARS-CoV, HIV and influenza (Wang et al., 2020a). In addition, ACE2 expression has been shown to vary between different populations, driven in part by extract expression quantitative trait loci (eQTLs) (Cao et al., 2020).

Several studies have shown that *ACE2* coding variants and eQTLs can influence the host susceptibility or resistance to SARS-CoV-2 infection, by altering S protein binding or ACE2 expression. However, many of these studies are based on *in silico* predictions from public datasets or have relatively few SARS-CoV-2 positive patient sample sizes, of which there is often limited diversity (Benetti et al., 2020; Novelli et al., 2020; Yildirim et al., 2021).

To identify population-level variants that might contribute to SARS-CoV-2 infection differences, we analysed *ACE2* variants within the 100,000 Genomes Project (100KGP). This dataset consists of whole-genome sequencing (WGS) data from patients recruited through the National Health Service (NHS) representing the diverse population of the United Kingdom (United Kingdom) (100,000 Genomes Project, 2019), including 1,837 SARS-CoV-2 positive patients and 37,207 unrelated individuals with no record of SARS-CoV-2 infection, aggregated by five continental ancestries. Using this dataset, we provide evidence that *ACE2* variants correlate with COVID-19 susceptibility and severity, with population-specific effects.

Our findings provide a structure-function predictive framework for exploring genotype-phenotype correlations, and a genetic trait score to estimate individual liability to disease.

## Materials and Methods

### Patient Subjects

The 100KGP was established to sequence 100,000 genomes from around 85,000 NHS patients in the United Kingdom affected by a rare disease, cancer, or infectious disease, with detailed phenotype and clinical data (https://www.genomicsengland.co.uk/) (100,000 Genomes Project, 2019). Combining WGS data with medical records has created a ground-breaking research resource enriched for genetic diseases. As part of its recruitment target, Genomics England has highlighted the importance of engaging with the UK’s increasingly diverse black and minority ethnic population, who are underrepresented in clinical studies and research.

This dataset consisted of 6,274 patients who were tested for SARS-CoV-2, of which 1,837 tested positive. Of the positive cases, 1,411 (77%) were classified as not hospitalised, and 426 (23%) were classified as inpatients or deceased. We also used WGS data from a large control group consisting of 37,207 unrelated individuals from 100KGP (males = 17,066; females = 20,141; total allele number = 57,348), to scan for *ACE2* variants and eQTL distribution in different populations.

### Patient and Public Involvement

In accordance with the 100KGP, the 13 NHS Genomic Medicine Centres have established Patient and Public Involvement Network with leads that work together with representatives from Genomics England and NHS England to curate informed consent and the literature development surrounding the data used within this study. Patients and carers also play a role in the Ethics Advisory Committee for data used within this study. In addition, patients and carers are part of a participant panel that help to assure the data access process has the interests of participants as a central goal, through involvement in the data access review committee, Genomics England Clinical Interpretation partnership board, and ethics advisory committee. As part of a public engagement program Genomics England also can disseminate the results of this study through seminars, in an open-to-everyone and free-to-the-public setting.

### Ancestry Inference

Using multi-sample VCFs for participants in the 100KGP cohort, we generated Principal Components (PCs), calculated pairwise relatedness among samples, and estimated probabilities of genetic ancestry for five broad superpopulations. Broad genetic ancestry was estimated using ethnicities from the 1,000 genomes project phase 3 (1KGP3), by generating PCs for 1KGP3 samples and projecting all 100KGP participants onto these. The five broad superpopulations are European, South Asian, African, East Asian, Ad Mixed American, and multiracial individuals, labelled as Other.

### Classification of Hospitalised Status

Patient hospitalised status is based on whether the collected specimen was from an acute (emergency) care provider, an accident and emergency department, an inpatient location, or a result of health care associate infection. “Not hospitalised” is interpreted as there is no evidence that the patient has been an inpatient in the microbiological record, but this may still be a possibility as the microbiology data source is not linked to admissions data.

### Computational Modelling of Angiotensin-Converting Enzyme 2

We employed a computational method to predict the effect of the coding variants identified in the 100KGP dataset, based on homology modelling and computational docking of the virus-receptor protein-protein interaction. Several crystal structures of the SARS-CoV-2 RBD and ACE2 have been reported (pdb identifiers: 6VW1 (Shang et al., 2020), 6LZG (Wang et al., 2020b), 6M0J (Lan et al., 2020) 7A94 (Benton et al., 2020)) allowing us to demonstrate the utility of this model. Amino acids of interest were mutated in Coot (Emsley et al., 2010) and visually analysed using UCSF Chimera (Pettersen et al., 2004) Mutations were ranked according to their potential to affect protein stability and activity. Structure figures were prepared in Chimera.

### Public Gene Expression Dataset Acquisition and Analysis

We obtained expression data through the genotype-tissue expression (GTEx) database (https://gtexportal.org/home/) (Lonsdale et al., 2013) This data was also used to extract expression quantitative trait loci (eQTLs) for ACE2 (all data based on RNA-seq experiments). Population allele frequencies were obtained from 100KGP.

### Coding Variant Annotation and Function Prediction

The raw list of SNVs and indels among cases and controls were annotated using ANNOVAR (Wang et al., 2010). Variants in splicing regions, 5′-UTR, 3′-UTR, and protein-coding regions, such as missense, frameshift, stop loss, and stop gain mutations, were considered. *In silico* prediction of pathogenicity was assessed using PolyPhen2 (Adzhubei et al., 2013), CADD (Rentzsch et al., 2019), REVEL (Ioannidis et al., 2016) and GERP++ (Petrovski et al., 2015) scores. The highest priority variants were nonsynonymous or in the splice site, with a corresponding REVEL score above the default threshold of 0.5, a GERP++ score greater than two, and PolyPhen2 prediction outcome of “probably damaging”.

### Statistical Analysis

The data were analysed using chi-square tests to identify instances where there were differences between any of the groups (with Fisher’s exact where any expected value <5). Statistically significant differences were followed up with z-tests to compare proportions between pairs of groups and identify where the group differences were located (with Bonferroni correction). Statistical significance was considered at the 5% level (two-tailed).

## Results

### Summary of SARS-CoV-2 Positive Cases

**FIGURE 1:**
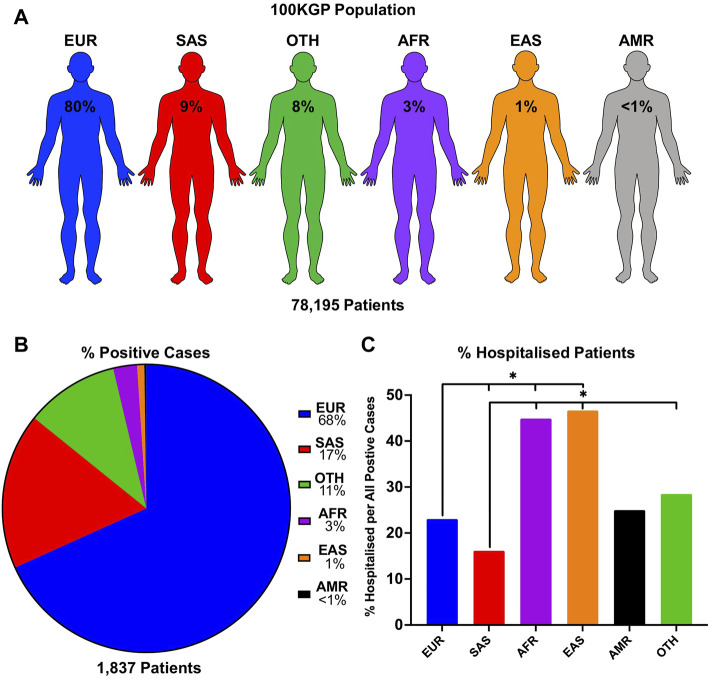
SARS-CoV-2 infection numbers and hospitalisation rate per ethnic group in the United Kingdom population. **(A)** The proportion of individuals in each population of the entire 100KGP cohort was assigned with a probability of >0.8 for any one ancestry. **(B)** Percentage of patients who tested positive for SARS-CoV-2 presented as positive cases in each ethnic background relative to the total positive cases in the 100KGP. **(C)** Percentage of hospitalised patients per total positive individuals in each ethnic group. *p* values are calculated using the Chi-square test.

To see if there is an observable difference in SARS-CoV-2 positivity and disease severity in different ethnic backgrounds, we used a unique dataset containing genetic information within the 100KGP. This dataset includes the SARS-CoV-2 test results, genetically predicted ancestry, and hospitalisation status of each patient. We stratified positive cases in the 100KGP by the five predominant superpopulations representing the general population in the United Kingdom (Figure 1A). Our data shows that 1,254 individuals of European (68.3%), 322 of South Asian (17.5%), 49 of African (2.7%), 15 of East Asian (0.8%), 4 of Ad Mixed American (0.2%) background, as well as 193 uncategorised individuals, labelled as Other (10.5%), tested positive for SARS-CoV-2 (Figure 1B).

The chi-square test was used to compare proportions of inpatients, demonstrating that hospitalised outcomes in SARS-CoV-2 positive patients were statistically different across ethnic groups (χ^2^ = 129.64, df = 5, *p* < 0.001). Individuals of East Asian and African background were approximately 50% more likely to be hospitalised compared to Europeans. Furthermore, the largest difference in hospitalisation rate was found between South Asians (16.2% hospitalised) and East Asians (46.8% hospitalised) (Figure 1C; Supplementary Table S1), suggesting that individuals of South Asian decent are less likely to be hospitalised from SARS-CoV-2 infections. In addition, Ad Mixed Americans did not statistically differ between any ethnic group, which may be due to the small sample size (*n* = 4). Nonetheless, these data provide evidence for varying degrees of susceptibility to COVID-19 in different ethnicities.

We hypothesise that these findings may reflect an underlying biological driver for viral susceptibility in different genetic backgrounds. To investigate this, we interrogated the 100KGP patient database for correlations between SARS-CoV-2 positivity status and genetic variants that alter *ACE2* protein structure, as well as expression. Although many mechanisms are involved in SARS-CoV-2 entry and host responses, we focused on ACE2 because it is the primary host receptor for viral entry and a limiting factor for infection.

### Angiotensin-Converting Enzyme 2 Coding Variants in 100KGP Controls

**FIGURE 2:**
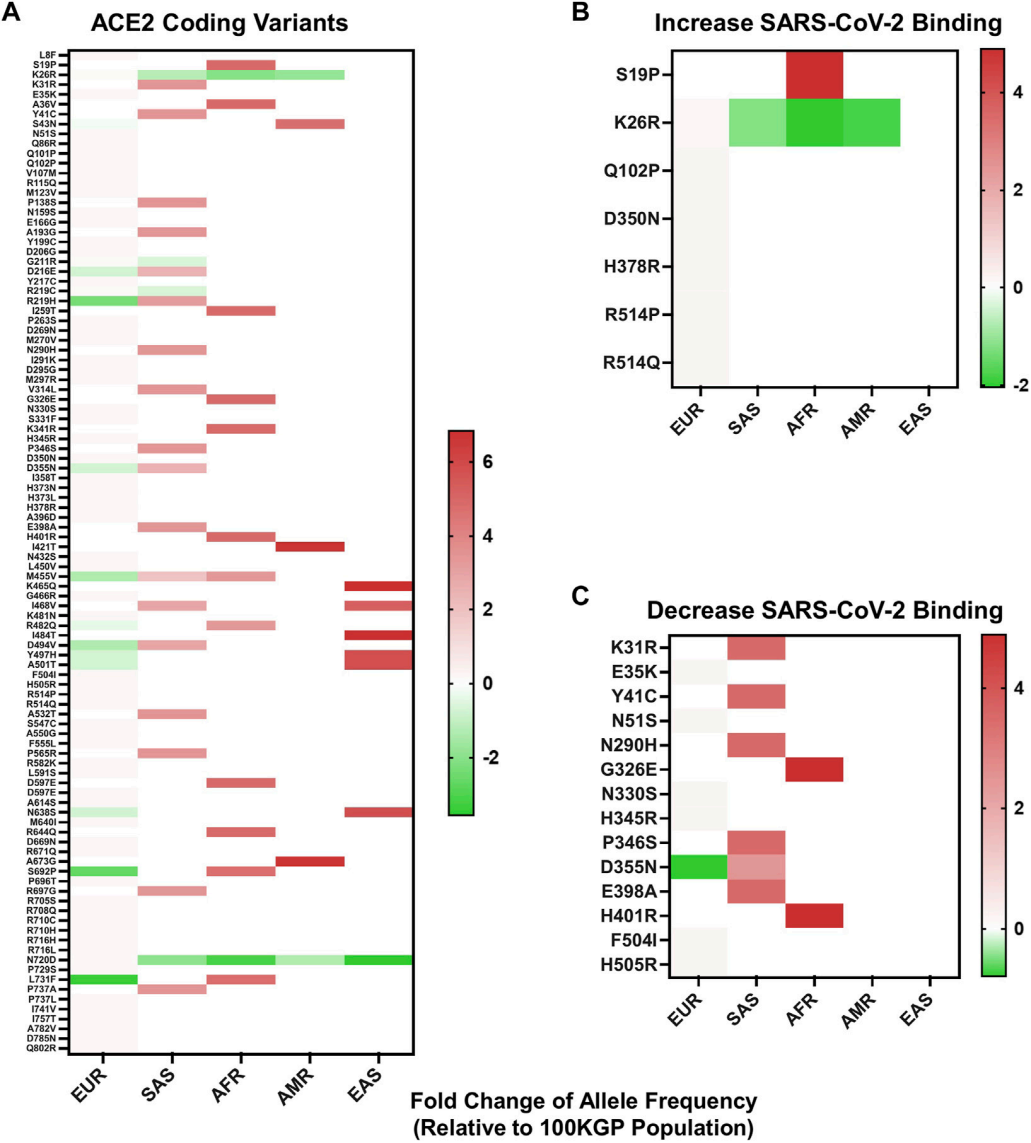
Allelic frequency of nonsynonymous ACE2 variants in different ethnicities. **(A)** Allelic frequency of *ACE2* coding variants present in individual ethnicities, graphed as a log base 2-fold change relative to the allelic frequency in 100KGP unrelated controls (n = 37,207). **(B)** Allelic frequency of *ACE2* coding variants in individual ethnicities that increase SARS-CoV-2 S protein binding affinity for ACE2. **(C)** Allelic frequency of *ACE2* coding variants in separate ethnicities that decrease SARS-CoV-2 S protein binding affinity for ACE2. All heat maps are graphed with values below and above zero set relative to the dataset. Shades of red indicate increased allelic frequency and green decreased allelic frequency, with lighter shades appearing as values move closer to no appreciated change relative to the 100KGP allelic frequencies, as indicated by white. Coding variants displayed by protein location and amino acid change.

To identify whether variants within *ACE2* could have a predicted influence over S protein-host cell interactions, we first identified coding variants within 37,207 unrelated individuals in the 100KGP dataset. This dataset represents the ethnic diversity of the United Kingdom population. Coding variants were stratified by the five predominant superpopulations represented within the United Kingdom population (European, South Asian, African, Ad Mixed American, and East Asian descent) and by allelic frequency. The resulting analysis provided 114 SNVs (Figure 2A), three deletions, and one insertion leading to a frameshift identified in one individual of European descent (Supplementary Table S2).

To see if any of these variants have the potential to affect binding of S protein, we compared our findings against data from a high-throughput functional mutagenesis screen that used deep sequencing to assess S-protein binding abilities to 2340 ACE2 variants (Chan et al., 2020) In the 100KGP control dataset, we identified 21 *ACE2* nonsynonymous variants that alter S protein binding, seven that increase binding and 14 decrease binding (Figures 2B,C **)**. We stratified these variants based on allelic frequency within different ethnic backgrounds. Populations of African and South Asian descent generally had the greatest number of alleles with increased frequency, relative to the general population (Figure 2A). This was also reflected in variants that both increase and decrease S protein binding affinity for ACE2 (Figures 2B,C). Specifically, we detected a greater frequency of variants in South Asians that decrease SARS-CoV-2 S protein binding affinity for ACE2 (Figure 2C), and one variant enriched within African descent populations that increases S protein-binding affinity for ACE2 (Figure 2B).

### Angiotensin-Converting Enzyme 2 Coding Variants in SARS-CoV-2 Positive Patients

To see if any coding variants in ACE2 correlate with decreased or increased propensity for SARS-CoV-2 positivity, we analysed WGS data of 1,837 SARS-CoV-2 positive patients and identified 18 nonsynonymous *ACE2* variants (Table 1; Supplementary Table S3). Six nonsynonymous variants were novel (p.V316L, p. H345R, p. H401R, p.M455V, p. Y515N, p. P737L), of which three are predicted to be deleterious based on *in silico* predictions. Three variants (p.K26R, p. H378R, p. Y515N) found across the interaction surface were shown to alter receptor affinity for the viral S protein based on deep mutagenesis experiments (Chan et al., 2020). Variants p. H378R and p. K26R increased binding, whereas the novel p. Y515N variant decreased binding.

**TABLE 1:** Characterisation of nonsynonymous ACE2 variants in SARS-CoV-2 patients. Intronic, intergenic and synonymous variants are considered non-pathogenic and therefore excluded from the list. The index score is based on the type of variant, the REVEL score, and the GERP++ score. An index score of 1 signifies a nonsynonymous variant with a corresponding REVEL score <0.5, suggesting low pathogenicity. An index score of 2 indicates a nonsynonymous variant with a REVEL score of >0.5, GERP++ score >2, CADD score of >20 and PolyPhen2 prediction outcome of “probably damaging”, suggesting high pathogenicity. Six variants were not reported in public databases (p.V316L, p.H345R, p.H401R, p.M455V, p.P737L, p.Y515N). Deep mutagenesis experimental data show p.K26R, p.H378R and p.Y515N altered binding affinity with S protein (Chan et al., 2020). This is consistent with our protein interaction analysis. Mutations are ranked according to their potential to affect protein stability and activity, denoted by asterisks (* = low, ** = medium, *** = high).

Variant	rsID	AA change	Index	Protein modelling	S Protein binding affinity in RBD	Minor allele frequency within 100KGP					
						Combined	EUR	SAS	AFR	AMR	EAS
chrX:15564123G > A	novel	p.P737L	1	—	NA	1.74E-05	2.03E-05	0	0	0	0
chrX:15564142G > A	rs147311723	p.L731F	1	—	NA	6.63E-04	6.09E-05	0	1.81E-02	0	0
chrX:15564175T > C	rs41303171	p.N720D	1	—	NA	2.34E-02	2.64E-02	6.09E-03	2.58E-03	9.21E-03	1.98E-03
chrX:15566293A > G	rs149039346	p.S692P	**2**	—	NA	4.06E-05	0	0	6.71E-03	0	0
chrX:15572271C > T	rs763593286	p.A532T	1	*	NA	5.23E-05	0	5.89E-04	0	0	0
chrX:15572322A > T	novel	p.Y515N	**2**	**	Decrease	0	0	0	0	0	0
chrX:15573419A > G	rs961921482	p.Y497H	1	*	NA	3.49E-05	2.03E-05	0	0	0	1.98E-03
chrX:15575745T > C	novel	p.M455V	1	**	NA	5.23E-05	2.03E-05	1.96E-04	5.17E-04	0	0
chrX:15578124A > G	rs1352508510	p.I421T	1	*	NA	1.74E-05	0	0	0	1.84E-03	0
chrX:15578184T > C	novel	p.H401R	**2**	**	Negligible	1.74E-05	0	0	5.17E-4	0	0
chrX:15578253T > C	rs142984500	p.H378R	**2**	***	Increase	1.92E-04	2.23E-04	0	0	0	0
chrX:15581257T > C	novel	p.H345R	**2**	**	Negligible	1.74E-05	2.03E-05	0	0	0	0
chrX:15581269T > C	rs138390800	p.K341R	1	*	NA	2.27E-04	0	0	6.71E-03	0	0
chrX:15581345C > A	novel	p.V316L	1	*	NA	1.74E-05	0	1.96E-04	0	0	0
chrX:15587768G > A	rs200745906	p.P263S	**2**	*	NA	1.92E-04	2.23E-04	0	0	0	0
chrX:15589385G > A	rs372272603	p.R219C	1	*	NA	9.42E-04	1.04E-03	5.89E-04	0	0	0
chrX:15589409C > T	rs148771870	p.G211R	1	*	NA	2.14E-03	2.35E-03	1.37E-03	0	0	0
chrX:15600835T > C	rs4646116	p.K26R	1	*	Increase	6.42E-03	7.10E-03	2.75E-03	1.55E-03	1.84E-03	0

**FIGURE 3:**
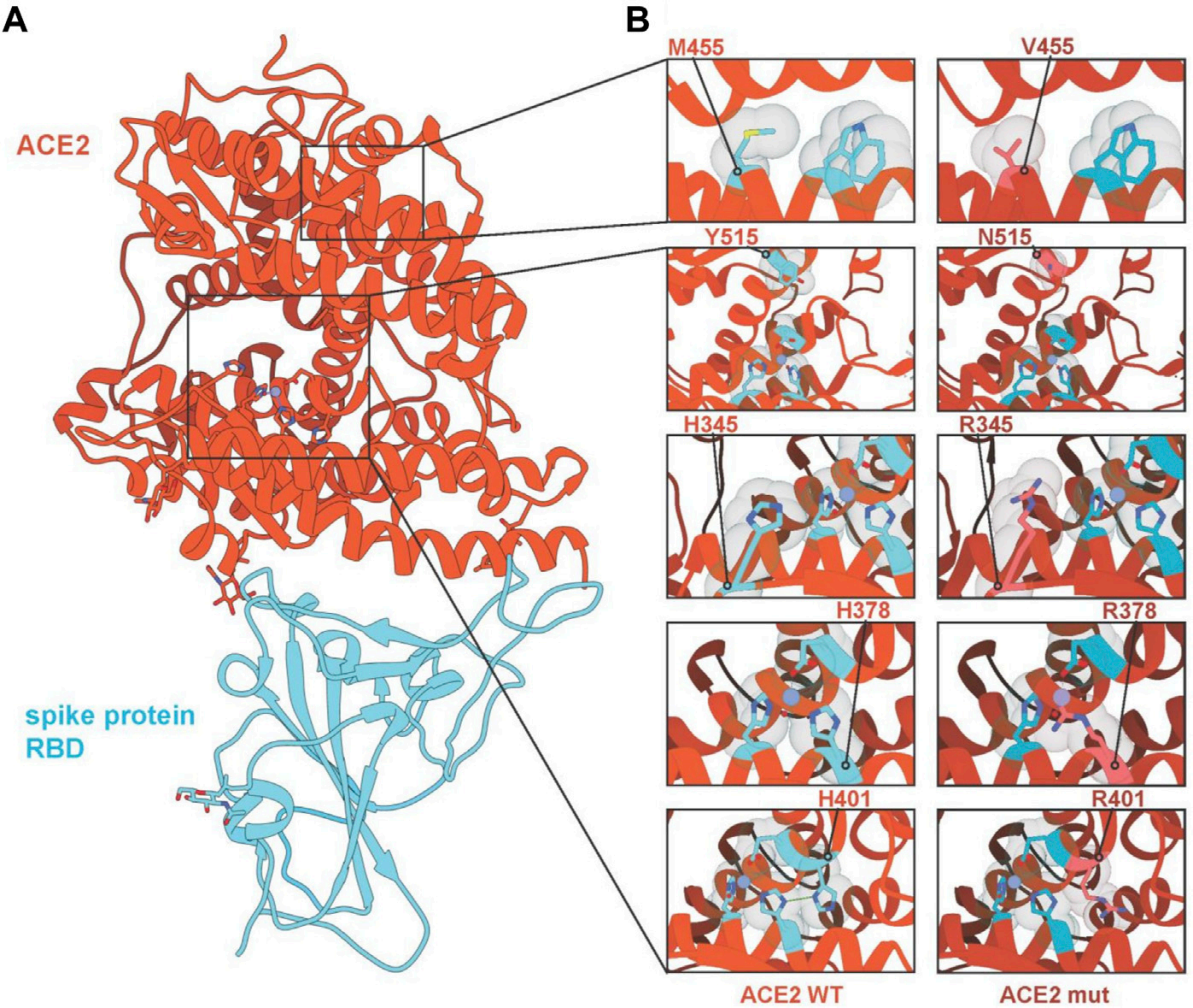
Interaction diagrams of ACE2 variants and SARS-CoV-2 S-protein. **(A)** Crystal structure of ACE2 and SARS-Cov-2 S protein (pdb identifier: 6LZG) with ACE2 coloured in red-orange and the RBD domain of the SARS-CoV-2 S protein in light blue. Regions of interest are highlighted and expanded in panel **(B)**, showing mutated residues M455V, Y515N, H345R, H378R and H401R with wild-type sequences (left) and mutated sequences (right). Hydrogen bonds are shown in green, Zinc ions are shown in purple, and water molecules are shown in red.

In addition, we analysed the effect of these variants on protein structure and assessed if they might alter the interaction between ACE2 and the SARS-CoV-2 S protein (Figure 3). While predicting the effect of single mutations in the core of the protein on the interaction of ACE2 with the S protein is challenging, we can nevertheless make predictions on the impact on protein stability. We selected five mutations for structural analysis (H345R, H378R, H401R, M455V and Y515N). H378R is the mutation with the most severe destabilisation effect on ACE2 as it directly coordinates the Zn^2+^ ion bound by the HEXXH + E motif of the ACE2 metallopeptidase. Similarly, H401 disrupts an H-bond between H378 and H401, destabilising the motif, likely to a lesser extent. H345R does not interact directly with either of the amino acids that coordinate the Zn^2+^ ion, but its substitution with arginine could have a negatively affect. Y515 can be found lining the pocket providing access to the Zn^2+^ motif and mutations could interfere with potential interaction partners. S-aromatic motifs have been reported to have stabilising effects on proteins (Aledo, 2019); thus, substituting methionine for valine at position 455 can negatively affect protein stability as M455 interacts with W477 through an S-aromatic interaction (Figure 3).

### Angiotensin-Converting Enzyme 2 Splice Variants in SARS-CoV-2 Positive Patients

We also identified an *ACE2* splice region variant (rs2285666 C/T) that was significantly more prevalent among SARS-CoV-2 patients than the control population (*p* = 0.015). Further, intra-ethnic comparisons showed that this variant was more prevalent in inpatients (minor allele frequency (MAF) = 23.9%) compared to patients not hospitalised (MAF = 18.4%) within the European population (*p* = 0.029), suggesting it plays a role in susceptibility and severity (Supplementary Table S4).

The rs2285666 C/T SNP is located on the fourth base position of intron three, which may affect gene expression via mRNA alternate splicing mechanisms (Yang et al., 2015). According to the HaploReg tool (v4.1), rs2285666 alters transcription factor binding sites; namely, HNF1, and Ncx motifs. Using an ELISA method, Wu et al. (2017) showed that the rs2285666 SNP elevated serum ACE2 levels, with the T/T genotype increasing expression by almost 50%. Similarly, the GTEx database classifies this variant as a significant eQTL, which is associated with an increased expression of ACE2, CA5BP1, PIR and TMEM27, and decreased expression of CA5B.

Of note, previous studies have identified rs2285666 as a risk factor for hypertension, type 2 diabetes, and coronary heart disease (Roach and Bugan, 2014; Asselta et al., 2020). Taken together, this polymorphism may increase SARS-CoV-2 infection rates by altering ACE2 expression and predisposing to comorbidities observed in COVID-19 patients.

We also analysed *ACE2* variants in the 3′UTR, 5′UTR and promoter regions, where the promoter is defined as 2 kb upstream of the start codon. We found no significant differences in the allele frequencies between SARS-CoV-2 positive patients and controls, or patients stratified by hospitalised status. However, when examining intra-ethnic differences in allele and haplotype frequencies, the rs199651576 T/C 5′UTR polymorphism was found to be higher in SARS-CoV-2 patients (MAF = 0.12%) compared to controls (MAF = 0.016%) within the European population (*p* = 0.039) (Supplementary Table S4). The function of this variant remains unclear, although it may be involved with the translational regulation of the ACE2 mRNA transcript.

### Inter-Ethnic Differences in Angiotensin-Converting Enzyme 2 Coding Variants

To see if any of the variants found within SARS-CoV-2 positive patients differ between ethnicities, we compared the MAFs from 37,207 unrelated individuals, representing the general population of the United Kingdom Z-test comparisons were made between ethnic groups (with Bonferroni correction where there were more than two groups). The chi-square test was used to see whether distributions of variables differ from each other. Variants p. K26R (χ^2^ = 23.12, df = 3, *p* < 0.001) and p. N720D (χ^2^ = 137.55, df = 4, *p* < 0.001) were statistically different between groups. These variants were also the most frequent in the 100KGP cohort and Genome Aggregation Database (gnomAD) (Karczewski et al., 2020).

The p. K26R variant was more common in Europeans (MAF = 0.710%) compared to South Asians (MAF = 0.275%; *p* < 0.001) and Africans (MAF = 0.155%; *p* = 0.001). The K26 residue lies on the proximal end of the SARS-CoV-2 RBD–ACE2 interface (Lan et al., 2020). Structural predictions have shown that the K26R variant enhances the affinity of ACE2 for SARS-CoV-2 by strengthening the hydrogen bond between H34 and Y453 of the S-protein (Stawiski et al., 2020; Al-Mulla et al., 2021). This is consistent with deep mutagenesis experimental data using a synthetic human ACE2 mutant library (Chan et al., 2020).

Variant p. N720D was also more common in Europeans (MAF = 2.64%) compared to South Asians (MAF = 0.609%; *p* < 0.001), Africans (MAF = 0.285%; *p* < 0.001) and East Asians (MAF = 0.198%; *p* < 0.001). The p. N720D variant lies in the C-terminal collectrin domain of ACE2. Although it is not involved in the SARS-CoV-2 S-protein interaction, studies have shown that it enhances transmembrane protease, serine 2 (TMPRSS2) binding and cleavage of ACE2. As TMPRSS2 cleavage of the ACE2 receptor increases viral entry, the p. N720D variant may lead to increased COVID-19 susceptibility (Mohammad et al., 2020; Al-Mulla et al., 2021).

Finally, the p. Y497H variant was more prevalent in East Asians (MAF = 0.198%) compared to Europeans (MAF = 0.00203%) (*p* < 0.001), but absent in other populations (Table 1; Supplementary Table S5).

### Angiotensin-Converting Enzyme 2 eQTLs in SARS-CoV-2 Positive Patients

While protein-protein interactions can explain some components of viral entry and potential susceptibility in variant carrying at-risk groups, differential expression of ACE2 may also be a contributor to viral-host cell interactions.

Among the 31 GTEx human tissues, the highest ACE2 expression levels are found in the testis, small intestine, kidneys, heart, thyroid, and adipose tissue (Lonsdale et al., 2013). The lowest ACE2 expression levels are found in whole blood, spleen, brain, and skeletal muscle. The lungs, pancreas, oesophagus (muscularis) and liver had intermediate ACE2 expression levels.

The Human Protein Atlas (HPA) (Thul and Lindskog, 2018) database shows that ACE2 had relatively high expression levels in the duodenum, small intestine, gallbladder, kidneys, testis, seminal vesicle, colon, rectum, and adrenal gland. The HPA database also showed that the gastrointestinal tract (duodenum, small intestine, colon, and rectum), kidney, gallbladder, and male tissues (testis and seminal vesicle) had high expression levels of both the ACE2 protein and gene. Interestingly, the results obtained from the HPA (fresh frozen tissue) and GTEx (postmortem tissue) are similar, suggesting negligible effects of the sampling procedures used by the GTEx consortium on RNA degradation.

We also interrogated the GTEx database for the distribution of ACE2 eQTLs (GTEx analysis Release V8). For ACE2, we identified 288 significant (FDR <0.05) eQTLs in 20 different tissues, including, adipose (*n* = 31; 10.8%), brain (*n* = 246; 85.4%), testis (*n* = 2; 0.7%), and prostate (*n* = 60; 20.8%). It would be valuable to compare the frequency of eQTL variants with ACE2 expression specifically in the lung with susceptibility to viral infection and severity of COVID-19. However, to date, no eQTL for ACE2 in the lung has been reported in the GTEx database, thus warranting further investigations in this regard.

**FIGURE 4:**
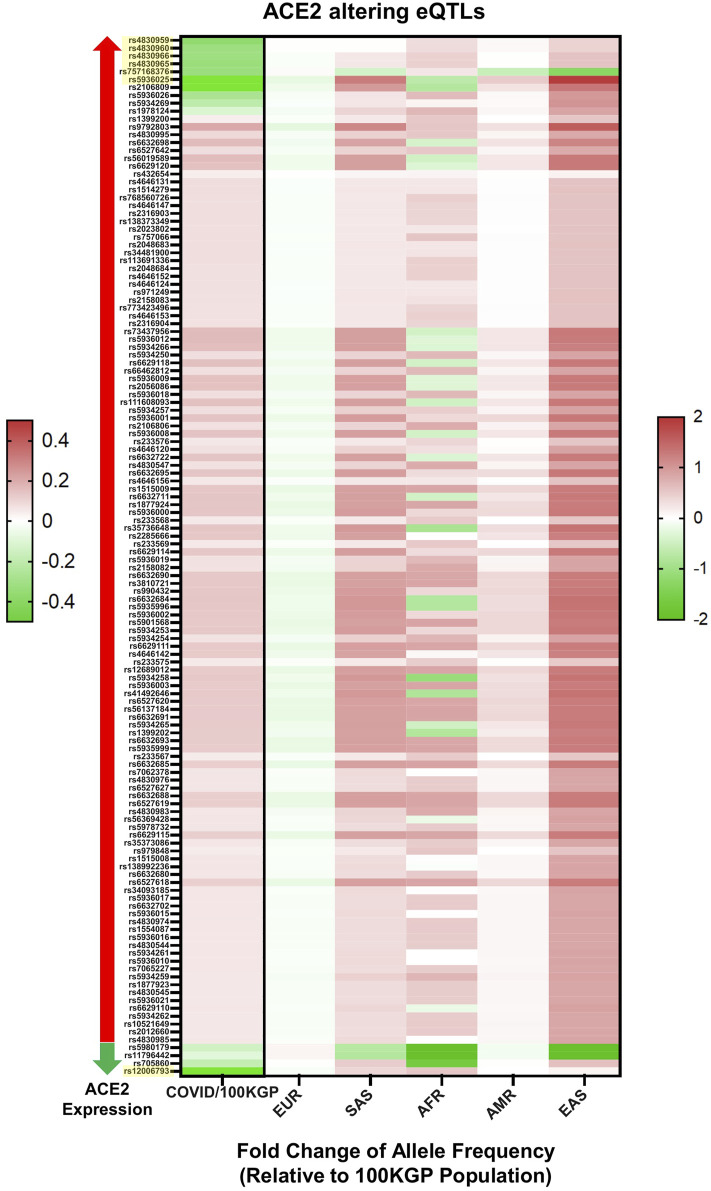
Statistically significant eQTLs in SARS-CoV-2 positive individuals. Displayed by total positive individuals (value on the left) and by ethnic background (value on the right), values are presented as log base 2-fold change relative to the allelic frequency within non-positive individuals found in the 100KGP (n = 37,207). eQTLs are also subdivided by effects on ACE2 expression, with eQTLs known to associate with increased ACE2 expression marked by a red arrow and those associated with decreased ACE2 expression marked by a green arrow. All displayed eQTLs are significant based on chi-squared test, and yellow highlighted eQTLs had Cohen’s values above 0.2. All eQTLs are referenced by rs identifiers.

The allele frequencies of the 288 eQTL variants were compared among different ethnicities in the 100KGP dataset (Figure 4; Supplementary Table S6). Across this cohort, we performed a case-control genetic association analysis on ACE2 eQTLs. Of the 288 eQTLs associated with tissue expression of ACE2 in the GTEx database, 132 were significant between patients and controls. Twenty-three were significantly different after Bonferroni correction, seven of which had a Cohen’s effect size, d, of >0.2. The largest MAF difference was observed in rs12006793 (d = 0.91). This eQTL decreases ACE2 expression and was more common in the control group (Supplementary Table S7,S9), suggesting it reduces COVID-19 risk. This eQTL was statistically different between ethnic groups (χ^2^ = 801.06, df = 4, *p* < 0.001). The eQTL prevalence significantly differed between all ethnic comparisons, except between Ad Mixed Americans and East Asians, and Ad Mixed Americans and Europeans. It was more common in Africans (MAF = 66.6%) and South Asians (MAF = 60.1%). However, according to GTEx, it was only expressed in adipose tissue (omentum) with a normalised enrichment score of -0.1.

When comparing hospitalised SARS-CoV-2 positive patients with patients not hospitalised, only two eQTLs were statistically significant after multiple testing correction, but none had a Cohen’s effect size greater than 0.2. Interestingly, however, rs12006793 was one of the two significantly enriched among the patients stratified by hospitalised status (Supplementary Table S8). Based on this data, rs12006793 may be an important marker for COVID-19 susceptibility and severity.

## Discussion

Based on the hypothesis that a predisposing genetic background may contribute to the observed clinical variability of COVID-19, we set out to investigate whether variation in *ACE2* might modulate susceptibility to SARS-CoV-2 and influence severity. While large-scale GWAS have been conducted on COVID-19 patients (Ellinghaus et al., 2020; Hu et al., 2021; Niemi et al., 2021; Pairo-Castineira et al., 2021), our study included investigating rare susceptibility alleles, which are often excluded in GWAS. Further, genetic variation in *ACE2* regulatory regions is a strong candidate for identifying differences in ACE2 activity. Yet, studies on genotype-dependent ACE2 expression in SARS-CoV-2 patients are currently lacking.

We systematically analysed *ACE2* polymorphisms associated with higher protein expression and coding-region variants between patients and controls, as well as individuals from different ethnicities. We provide evidence for differences in coronavirus S protein binding affinity between ACE2 variants in different ethnicities and eQTLs significantly associated with COVID-19 susceptibility.

Among 1,837 SARS-CoV-2 positive patients, six nonsynonymous *ACE2* variants (V316L, H345R, H401R, M455V, Y515N, and P737L) are not included in public databases, such as gnomAD, dbSNP, and 1KGP3. Based on the functional analysis of a synthetic human ACE2 mutant library for RBD-binding affinity (Chan et al., 2020), p. Y515N reduces and p. K29R increases the receptor affinity for the viral S protein. Further, the top variants predicted to disrupt protein function based on the tools used in this study include p. P263S (rs200745906), p. H345R (novel), p. H378R (rs142984500), p. H401R (novel), p. Y515N (novel), and p. S692P (rs149039346). Some of these damaging ACE2 missense variants may be protective against SARS-CoV-2 infection by either disrupting the proteolytic activation of SARS-CoV-2 or decreasing S-protein binding.

Two missense variants (K26R and N720D) were statistically more prevalent in the European population. We postulate that K26R increases the affinity of ACE2 for SARS-CoV-2, while N720D enhances TMPRSS2 activation and subsequent viral entry.

By performing structural modelling of mutations, we show that the H378R variant, which mutates one of the Zn^2+^ binding histidines to arginine, could directly weaken the binding of catalytic metal ions and reduce ACE2 peptidase activity. Further, H401R is located very close to the Zn^2+^ binding pocket and could destabilise the ACE2 structure.

Another potential explanation for different prognostic outcomes in these patients may involve ACE2 receptor expression levels. While the function of the rs199651576 T/C 5′UTR SNP remains unclear, it may be involved with the translational regulation of the ACE2 mRNA transcript. The splice site variant rs2285666 enriched in SARS-CoV-2 positive patients relative to 100KGP controls, and hospitalised European patients relative to outpatients in intra-ethnic comparisons, increases ACE2 expression possibly via mRNA alternate splicing mechanisms (Yang et al., 2015). Studies have shown that rs2285666 correlates with hypertension, coronary heart disease and type 2 diabetes (Roach and Bugan, 2014; Asselta et al., 2020). This variant is more common among South Asians and East Asians and may help predict COVID-19 outcomes.

Twenty-three eQTLs for the *ACE2* gene were significantly different between patients and controls after multiple-comparison correction. Of these, ten with minor alleles that associated with higher tissue expression of ACE2 were associated with COVID-19 aetiology. Intriguingly, two eQTLs whose MAF was associated with lower tissue expression were associated with a reduced risk of testing positive for SARS-CoV-2. One of these polymorphisms (rs12006793) had a Cohen’s large effect of 0.91, suggesting it may be a predictive marker for reduced susceptibility.

These eQTLs have considerable differences in allele frequencies among ethnicities within the 100KGP dataset (Figure 4). Consistent with our findings, Cao et al. (2020) showed that the East Asian populations have much higher allele frequencies in the eQTL variants associated with higher ACE2 expression, suggesting population differences in response to SARS-CoV-2. In a study analysing single-cell RNA sequencing data of human lungs of eight donors, Zhao et al. (2020) showed that ACE2 was more abundantly expressed in type II alveolar epithelial cells of a male Asian donor than both Black and White donors.

**FIGURE 5:**
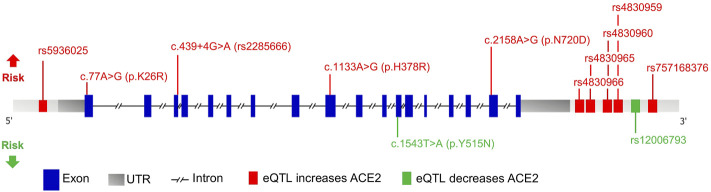
Schematic representation of the ACE2 gene with variants affecting risk. Genomic structure is based on the Ensembl canonical transcript (ENST00000252519.8), which has 18 exons, a transcript length of 3,339 bps, and a translation length of 805 residues. Exons are shown as blue boxes, the 5′ and 3′ UTRs are shown as grey boxes, and the horizontal black dashed line represents the introns. The eQTLs that increase and decrease ACE2 expression levels are depicted as green and red boxes, respectively. Only eQTLs with a Cohen’s d effect size >0.2 are shown. Missense and splicing variants are shown with their CDS and protein position. The locations of the variants associated with increased and decreased COVID-19 susceptibility are shown above and below the gene, respectively.

East Asians had a higher frequency of upregulating variants and lower frequency of downregulating variants than other populations. The resultant higher levels of ACE2 in this population may lead to higher COVID-19 susceptibility. Further, Africans showed a genetic predisposition for lower expression levels of ACE2, implicating the opposite. It is possible that the effect of individual eQTLs is too small to affect the receptor function of ACE2. However, genetic trait scores are increasingly used to predict phenotype based on a cumulative contribution of genetic factors to a specific trait. Herein we highlight variants of significance in COVID-19 patients as a means of refining estimates of individual liability to disease (Figure 5).

The 100KGP dataset shows a high proportion of participants testing positive for SARS-CoV-2 and hospitalised compared to the rest of the population. The higher hospitalisation rate could partly reflect increased interactions with the healthcare system, or those patients with pre-existing comorbidities are at increased risk of COVID-19. While this may not accurately represent the United Kingdom population, studying at-risk individuals offers a unique opportunity to investigate variability in COVID-19 outcomes. This may lead to improved prevention measures, triage strategies and clinical intervention.

In this study, we provide further insights into the potential role of genetics on SARS-CoV-2 infectivity and disease severity. We provide evidence of a genetic link between the *ACE2* genotype and COVID-19 disease severity and suggest that eQTLs and coding variants may inform COVID-19 risk stratification.

## Data Availability

All datasets generated for this study are included in the article and supplementary files. Primary data from the 100KGP database is held in a secure Research Environment and available to registered users. Requests to access the primary data should be directed to https://www.genomicsengland.co.uk/about-gecip/for-gecip-members/data-and-data-access.

## Abbreviations

100KGP, 100,000 Genomes Project; 1KGP3, 1,000 genomes project phase 3; ACE2, angiotensin-converting enzyme 2; COVID-19, coronavirus disease 2019; eQTLs, extract expression quantitative trait loci; GTEx, genotype-tissue expression (GTEx); GWAS, genome-wide association study; NHS, National Health Service; PC, principal component; RBD, receptor-binding domain; SARS-CoV-2, severe acute respiratory syndrome-coronavirus 2; SNP, single nucleotide polymorphism; UK, United Kingdom; WGS, whole-genome sequencing.
